# Effects of Melatonin on Intestinal Microbiota and Oxidative Stress in Colitis Mice

**DOI:** 10.1155/2018/2607679

**Published:** 2018-02-06

**Authors:** Dan Zhu, Yong Ma, Sujuan Ding, Hongmei Jiang, Jun Fang

**Affiliations:** College of Bioscience and Biotechnology, College of Animal Science and Technology, Hunan Agricultural University, Changsha, Hunan 410128, China

## Abstract

This study investigated the antioxidant capacity and intestinal bacteria community in a mouse model of DSS-induced colitis. Twenty mice were randomly assigned to two treatments: mice with colitis induced by 5% DSS (DSS group) and mice with colitis induced by 5% DSS that also received melatonin treatment (MEL group). The DSS group showed significantly less antioxidant capability than the MEL group, but the two groups did not differ significantly in terms of diversity index (Shannon and Simpson), bacterial culture abundance (Chao1 and ACE), and coverage (Good's coverage estimator). Bacteroidetes were the most abundant phylum in the DSS group (58.93%), followed by Firmicutes with 31.46% and Proteobacteria with 7.97%. In contrast, Firmicutes were the most abundant in the MEL group (49.48%), followed by Bacteroidetes with 41.63% and Proteobacteria with 7.50%. The results support the use of melatonin for prevention of intestinal bowel disease due to its modulatory effect on antioxidant capability and microbiota in mice with colitis. Melatonin was demonstrated to improve the oxidative stress resistance of mice with colitis and regulate the intestinal microbial flora, thus improving intestinal health.

## 1. Introduction

Inflammatory bowel disease (IBD) includes a group of high-prevalence conditions that occur worldwide, including Crohn's disease (CD) and ulcerative colitis (UC), both of which have had increasing incidence rates at the global level [1]. The development of IBD is underpinned by genetic and environmental factors. More than 200 loci related to risk of UC or CD have been distinguished via IBD genetic analyses [2–4]. A variety of environmental and behavioral factors such as diet, smoking, sleep patterns, hygiene, and usage of antibiotics contribute to IBD development [5], and the risk of IBD is also affected by the intestinal microbiota [6].

The microbial community assemblage (microbiota) of the gastrointestinal tract plays numerous critical roles in human physiology and metabolism, leading to the suggestion that the microbiota comprises a virtual superorgan [5]. The functions of the microbiota include extraction of indigestible nutrients from food that are otherwise inaccessible to humans; synthesis of vitamins; promotion of intestinal homeostasis via regulation of secretions and motility; and education of the immune system to develop mucosal tolerance. Mucosal tolerance is particularly important, because the main characteristic of IBD is poor control of the mucosal immune response due to a loss of mucosal tolerance, coupled with dysfunctional protective innate immunity to dysbiotic microbiota. In fact, all chronic diseases are related to intestinal flora, although the evidence of IBD's intestinal pathogenesis is currently strongest [7]. Metagenomic analysis of IBD microbiomes has demonstrated that 12% of genetic networks that comprise unique metabolic pathways are significantly increased or decreased, whereas only 2% of bacterial genera show significant changes in abundance relative to healthy controls. Amino acid biosynthesis and carbohydrate metabolism are among the identified metabolic pathways. Furthermore, the transformation process of this disease is unique and has specific pathways.

The direct result of inflammation and oxidative stress is harmful to proteins, lipids, DNA, and organelles. Inflammatory cells produce numerous cytokines and chemokines that generate reactive oxygen species (ROS) and reactive nitrogen species (RNS) in both phagocytic and nonphagocytic cells by activating protein kinase signaling [8]. There is ample evidence that the overproduction of ROS/RNS accompanies chronic intestinal inflammation. Animal and human experiments have shown that IBD pathophysiology is significantly underpinned by excessive ROS/RNS production leading to oxidative stress and redox modulation [9].

During the 1960s, melatonin (*N*-acetyl-5-methoxytryptamine) was isolated and chemically identified as an endogenous hormone synthesized and secreted by the pineal gland [10]. Several studies have demonstrated that UC is eased after oral administration of melatonin. The main mechanisms of melatonin are to decrease the abundance of matrix metalloproteinases [11, 12], regulate the attenuation of immunological damage by the activity of macrophages [13], decrease oxidative stress (which is signaled by elevated levels of lipid peroxides) [6], inhibit the production of nitric oxide [14], suppress the activity of nuclear factor-kappa beta (NF-kb), and decrease the level of cytokines that promote inflammation [15].

In short, ample evidence shows that melatonin possesses strong antioxidant activity [16, 17]. It may have use as an antioxidant in a range of diseases. Melatonin is more beneficial than other antioxidant molecules in some respects because of certain physical and chemical attributes [18]. In this study, a model of DSS-induced IBD was used to assess the effects of melatonin on oxidative stress and the intestinal microbiota. In the treated mice, the intestinal microbial community was changed and the antioxidant capacity increased. The improved antioxidant capacity can be linked to the altered population of gut microbes after treatment with melatonin. This study is the first to report the important role of melatonin in the inflammation of the colon.

## 2. Materials and Methods

### 2.1. Experimental Design

The experiment was initiated after approval was obtained from the Animal Care and Use Committee of Hunan Agricultural University and was performed in conformance to Chinese animal welfare standards. The experiment used 20 ICR mice (age, 8 weeks; weight, 20 ± 0.63 g) that were randomly divided into a DSS group and a MEL group. Ten mice per group was deemed sufficient to discern treatment effects. Colitis was induced in the mice in both groups by means of 5% DSS in water. The MEL group also received melatonin (5% DSS and 0.2 mg/L melatonin in water; the bottle of melatonin was wrapped in tin foil paper). A basal diet was given to all mice for 3 days before the experiment. Food and water were not restricted during the 1-week experiment, and conditions of 22°C to 24°C, 55% to 60% humidity, and 12-hour light/dark cycles were maintained, with the lights being turned on at 08:00. On the morning of the eighth day, after completion of the experiment, the mice were anesthetized by intraperitoneal injection of tribromoethanol (250 mg/kg body weight) and killed by cervical dislocation, and blood was then collected. The colon contents were also collected, frozen in liquid nitrogen, and stored at −80°C until needed.

### 2.2. Plasma Antioxidant Power

ABTS (2,2′-azino-bis(3-ethylbenz-thiazoline-6-sulfonic acid)) radical cation decolorization was used to measure the overall antioxidant capability in the plasma of mice subjected to the treatment described above. Before use, a 10 mM ABTS solution was mixed with 2 mM hydrogen peroxide, and the mixture was kept in storage overnight in the dark at 4°C. An absorbance of around 0.31 at 660 nm was achieved by diluting the ABTS^∙+^ solution (1 : 10). The absorbance was measured 5 minutes after the addition of 1000 *μ*L buffer (pH 3.6), 10 *μ*L plasma, and 25 *μ*L ABTS^∙^ in a cuvette. In every assay, a blank was run, and the measurements were performed three times. The results were expressed as the micromolar equivalent of the plasma antioxidant ascorbic acid [19].

### 2.3. Extraction of DNA and PCR Amplification

After extraction of microbial DNA from the colon contents, PCR amplification was applied to the V3-V4 region of the bacterial 16S ribosomal RNA gene (95°C for 2 minutes, followed by 25 cycles at 95°C for 30 seconds, 55°C for 30 seconds, and 72°C for 30 seconds, and a final extension at 72°C for 5 minutes). For the amplification, the primers 515F 5′-barcode- GTG CCA GCM GCC GCG G-3′ and 907R 5′-CCG TCA ATT CMT TTR AGT TT-3′ were used, with the barcode denoting a sequence of eight bases that was singular to every sample. The PCR reactions were conducted three times, with a 20 *μ*L mixture consisting of 4 *μ*L 5x FastPfu Buffer, 2 *μ*L 2.5 mM dNTPs, 0.8 *μ*L of each primer (5 *μ*M), 0.4 *μ*L FastPfu Polymerase, and 10 ng template DNA.

### 2.4. Sequencing of Illumina MiSeq and Sequencing Data Processing

The AxyPrep DNA Gel Extraction Kit (Axygen Biosciences, Union City, CA) was used in keeping with the manufacturer's guidelines to extract amplicons from 2% agarose gel and purify them. Meanwhile, QuantiFluor-ST (Promega, USA) was used for amplicon quantification. An Illumina MiSeq platform was used to pool the purified amplicons in equimolar and paired-end sequence (2 × 250) in keeping with the standard protocols. QIIME (version 1.17) permitted demultiplexing and filtering for quality of raw fastq files based on three criteria: (i) truncation of the 300 bp reads at all sites with an average quality score of less than 20 across a sliding window of 50 bp, while the truncated reads with a length of less than 50 bp were eliminated; (ii) barcodes were precisely matched, with mismatch of just two nucleotides in primer matching and elimination of reads with unclear characters; (iii) assemblage based on overlap sequence was performed solely in the case of sequences with overlaps of more than 10 bp. Reads were eliminated if it was not possible to assemble them. UPARSE (version 7.1, http://drive5.com/uparse/) was used to group together operational units (OTUs) with 97% similarity cut-off, while UCHIME permitted identification and elimination of chimeric sequences. The RDP Classifier (http://rdp.cme.msu.edu/) was used to taxonomically analyze every 16S rRNA gene sequence by comparison with the Silva (SSU115)16S rRNA database, at 70% confidence threshold [20].

### 2.5. Statistical Analysis

The SPSS 22.0 software was used for statistical analyses, and Student's* t*-test was applied for ABTS assay comparison. The data were expressed as the mean ± standard error of the means. Significance was attributed to values in the same row but with distinct superscripts (*P* < 0.05).

## 3. Results

### 3.1. Effects of Melatonin on Serum Oxidative Stress

**Figure 1:**
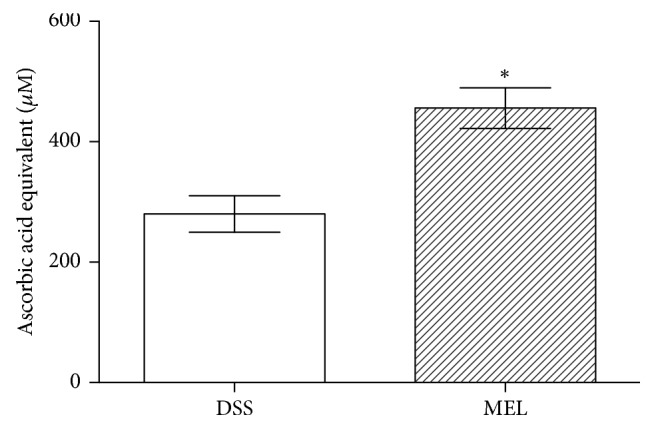
The antioxidant capacity in mice challenged with DSS after 7 days. DSS: control group challenged with DSS; MEL: 0.2 mg/L melatonin in the water and challenged with DSS. *∗* indicates the two groups differ significantly (*P* < 0.05).

The ABTS assay was performed to measure the plasma antioxidant capability. By comparison with the DSS group, the MEL group exhibited a significant increase in overall antioxidant capability (*P* < 0.05) (Figure 1).

### 3.2. The Diversity of Bacterial Communities in the Colon

In each colon sample, the 16S rRNA V3-V4 region was identified. Of the 163,950 viable sequences that were extracted, 83,229 were raw tags from DSS samples and 80,721 were raw tags from MEL samples. Once they were truncated, assembled, and filtered for quality, 71,741 DSS tags and 70,741 MEL tags remained relevant for analysis. The chosen sequences could suitably identify most parameters of bacterial diversity, on the basis of the normalized subsamples with 51,568 reads for each sample.  Table 1 provides the measured values of the diversity index (Shannon and Simpson), community abundance (Chao1 and ACE), and coverage (Good's coverage estimator), without any distinctions of statistical significance in these values.

### 3.3. Composition of Colon Bacterial Communities

**Figure 2:**
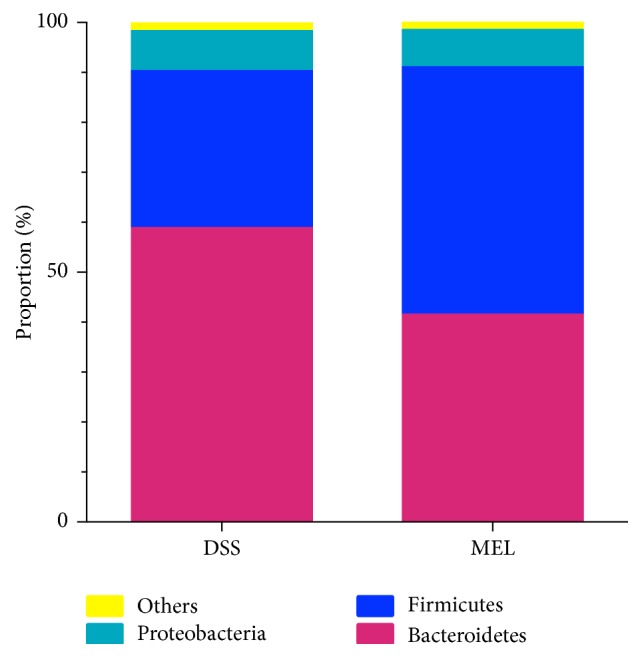
The dominant phylum in the colon of mice challenged with DSS after 7 days. DSS: control group challenged with DSS; MEL: 0.2 mg/L melatonin in the water and challenged with DSS.

Taxon-dependent analysis was conducted to evaluate the intestinal microbiota taxonomy. Feces were found to contain seven phyla, with Bacteroidetes, Firmicutes, and Proteobacteria being the most abundant (Figure 2). Bacteroidetes were the most abundant in the DSS group (58.93%), followed by Firmicutes with 31.46% and Proteobacteria with 7.97%. By contrast, Firmicutes were the most abundant in the MEL group (49.48%), followed by Bacteroidetes with 41.63% and Proteobacteria with 7.50%. The 15 genera with the greatest abundance are listed in Table 2. By comparison with DSS, MEL presented a greater abundance of Coprococcus_1 and Ruminococcaceae_UCG-014 (*P* < 0.05).

## 4. Discussion

In the human body, the largest immune organ is the intestine, which has the highest antibody production (40 mg/kg body weight/day) and includes 80% of all cells that produce antibodies [21, 22]. Around 10^14^ microbes from 400 to 500 species are present in the intestine [23]. Numerous host processes depend on the intestinal microbiota, such as breakdown of carbohydrates, regulation of assimilation of lipids from the diet, production of some vitamins and short-chain fatty acids, immunity development and support, gut motility regulation, and antipathogen protection. It is unsurprising then that a correlation has been established between microbiota dysbiosis and several disorders, such as IBD, obesity, and colon cancer. Furthermore, a central component of the intestinal mucosal barrier is the intestinal microflora. Hence, the mutual effect of the intestinal microflora and intestinal immunity is to be expected, as is the fact that their combined effect is felt in parts of the body other than the intestine. The seven major phyla of intestinal microbes are Firmicutes, Bacteroidetes, Actinobacteria, Proteobacteria, Verrucomicrobia, Cyanobacteria, and Fusobacteria [24], and* Bacteroides*,* Bifidobacterium*,* Clostridium*,* Eubacterium*,* Bacillus*,* Peptostreptococcus*,* Fusobacterium*, and* Ruminococcus* are the genera most prevalent in the colon [25].

The results of the experiment indicated that, compared with the DSS group, the MEL group had significantly higher levels not only of* Coprococcus*, but also of Ruminococcaceae, both of which are Firmicutes. Individuals with IBD are known to show poorer diversity of Firmicutes, which reflects the generally poorer diversity of the microbiota in the gut [26]. One study provided clear evidence that Firmicutes, particularly* Clostridium* groups, were less abundant in patients with UC and CD, whereas Proteobacteria were more abundant [26]. At the same time, numerous beneficial* Bacteroides*,* Eubacterium*, and* Lactobacillus* species were less abundant [26].

An earlier study using Google Earth Engine (GEE) and random forest analyses found that the genus* Coprococcus* occurred in lower levels in patients with IBD, particularly in nonresponders, than in control subjects. Indeed, for a while, agglutinating antibodies that target* Coprococcus* were taken as a CD screening biomarker [24]. In a different study, individuals with IBD showed a significant reduction in microbiota diversity, especially the abundance of* Lactobacillus* and* Bacteroides* [27]. By contrast, groups in our study did not differ significantly in terms of microbiota diversity, and the levels of* Lactobacillus* and* Bacteroides* were similar in both groups. However, despite such observations, it is not easy to establish whether IBD is caused by changes in gut microbiota or is the reason for those changes. One study compared CD and UC in terms of the transformations in several bacterial types and found that the two diseases were associated with significant reduction in the abundance of* Coprococcus*,* Roseburia*,* Faecalibacterium*, and* Ruminococcus* and in the overall diversity of the fecal microbiota, by comparison with healthy controls [28]. The features of the* Coprococcus* strain and* Eubacterium rectale *suggest that it could potentially be involved in IBD. That is, unlike other strains,* Eubacterium rectale* is capable of complement activation based on the alternative pathway and is particularly relevant for diagnosis, according to logistic discriminant analysis [29]. Furthermore, phagocytosis cannot be triggered by IgG antibodies that target only* Eubacterium rectale* [21]. Interestingly, a random forest analysis revealed that multiple genera of the Lachnospiraceae and Ruminococcaceae families, which were isolated from CD tissue samples, occurred in lower levels in nonresponders than in responders (especially Lachnospiraceae), despite not being involved in dysbiosis [30]. This finding calls for additional investigation of the potential involvement of members of these families in the pathophysiology of IBD. To conclude, our study has explored the extent to which the diversity and abundance of the microbiota in mice with colitis were affected by melatonin.

The signs of UC are oxidative stress and oxidative cell damage. Both are likely to play an important role in the pathogenesis of UC [31]. Pharmacological interventions in patients with UC have also demonstrated the importance of oxidative stress in this disease [32]. On one hand, melatonin can reduce oxidative damage, and, at the physiological level, it has been shown to stimulate gene expression of antioxidase enzymes or increase their activity [33]. Our study investigated the effects of antioxidant capacity in mice with colitis treated with melatonin and showed that melatonin can improve their antioxidant capacity. Interestingly, on the other hand, a previous study found that serum melatonin levels were significantly increased in mice treated with DSS [34]. This evidence supports our finding that melatonin has a highly beneficial therapeutic effect in mice with colitis.

## Figures and Tables

**Table 1:** Alpha diversity indices of fecal bacterial communities of mice.

Item	DSS	MEL
Raw tags	83229 ± 2407	80721 ± 2037
Effective tags	71741 ± 2107	70741 ± 1876
OTU	631 ± 50	587 ± 55
Shannon	5.54 ± 0.18	5.91 ± 0.15
Simpson	0.94 ± 0.01	0.96 ± 0.01
Chao1	632.29 ± 46.08	577.02 ± 48.47
ACE	654.22 ± 49.83	591.97 ± 53.67
Coverage	0.998 ± 0.000	0.998 ± 0.000

**Table 2:** Composition of fecal bacterial communities at the genus level of mice.

Phyla	Genus	DSS	MEL
Bacteroidetes	Alloprevotella	3.50 ± 0.84	2.16 ± 0.64
	Bacteroides	18.49 ± 2.96	11.76 ± 0.83
	Parabacteroides	2.23 ± 0.50	2.25 ± 0.96
Firmicutes	Anaerotruncus	0.62 ± 0.18	1.36 ± 0.57
	Coprococcus_1	0.52 ± 0.08^b^	1.95 ± 0.43^a^
	Erysipelatoclostridium	1.77 ± 0.57	2.24 ± 0.71
	Intestinimonas	0.53 ± 0.15	1.30 ± 0.39
	Lachnoclostridium	0.92 ± 0.23	0.63 ± 0.16
	Lachnospiraceae_NK4A136_group	7.82 ± 2.70	8.24 ± 2.48
	Lactobacillus	0.71 ± 0.23	0.89 ± 0.36
	Ruminiclostridium_9	1.55 ± 0.46	2.07 ± 0.57
	Ruminococcaceae_UCG-014	0.67 ± 0.05^b^	1.82 ± 0.20^a^
Proteobacteria	Desulfovibrio	0.70 ± 0.18	1.26 ± 0.30
	Helicobacter	4.80 ± 1.51	4.46 ± 1.97
	Parasutterella	0.79 ± 0.22	0.74 ± 0.29

The subscripts “a” and “b” indicate that the two groups differ significantly (*P* < 0.05).

## References

[1] Dolan K. T., Chang E. B. (2017). Diet, gut microbes, and the pathogenesis of inflammatory bowel diseases. *Molecular Nutrition & Food Research*.

[2] Jostins L., Ripke S., Weersma R. K. (2012). Host-microbe interactions have shaped the genetic architecture of inflammatory bowel disease. *Nature*.

[3] Huang C., Haritunians T., Okou D. T. (2015). Characterization of Genetic Loci That Affect Susceptibility to Inflammatory Bowel Diseases in African Americans. *Gastroenterology*.

[4] Liu J. Z., van Sommeren S., Huang H. (2015). Association analyses identify 38 susceptibility loci for inflammatory bowel disease and highlight shared genetic risk across populations. *Nature Genetics*.

[5] Ananthakrishnan A. N. (2013). Environmental risk factors for inflammatory bowel disease. *Journal of Gastroenterology and Hepatology*.

[6] Kostic A. D., Xavier R. J., Gevers D. (2014). The microbiome in inflammatory bowel disease: current status and the future ahead. *Gastroenterology*.

[7] Wu G. D., Bushmanc F. D., Lewis J. D. (2013). Diet, the human gut microbiota, and IBD. *Anaerobe*.

[8] Sánchez A., Calpena A. C., Clares B. (2015). Evaluating the oxidative stress in inflammation: role of melatonin. *International Journal of Molecular Sciences*.

[9] Bhattacharyya A., Chattopadhyay R., Mitra S., Crowe S. E. (2014). Oxidative stress: an essential factor in the pathogenesis of gastrointestinal mucosal diseases. *Physiological Reviews*.

[10] Stehle J. H., Saade A., Rawashdeh O. (2011). A survey of molecular details in the human pineal gland in the light of phylogeny, structure, function and chronobiological diseases. *Journal of Pineal Research*.

[11] Hardeland R. (2017). Melatonin signaling in T cells: Functions and applications. *Journal of Pineal Research*.

[12] Ren W., Wang P., Yan J. Melatonin alleviates weanling stress in mice: Involvement of intestinal microbiota. *Journal of Pineal Research*.

[13] Tork O. M., Amin S. N., Rashed L. A. Melatonin Reduces Cardiac Injury Induced by Lipopolysaccharides in Rats.

[14] Hagar H. H., El Medany A., El Eter E., Arafa M. (2007). Ameliorative effect of pyrrolidinedithiocarbamate on acetic acid-induced colitis in rats. *European Journal of Pharmacology*.

[15] Mazzon E., Esposito E., Crisafulli C. (2006). Melatonin modulates signal transduction pathways and apoptosis in experimental colitis. *Journal of Pineal Research*.

[16] Verma D., Hashim O., Jayapalan J., Subramanian P. (2014). Effect of melatonin on antioxidant status and circadian activity rhythm during hepatocarcinogenesis in mice. *Journal of Cancer Research and Therapeutics*.

[17] Gurer-Orhan H., Karaaslan C., Ozcan S. (2016). Novel indole-based melatonin analogues: Evaluation of antioxidant activity and protective effect against amyloid *β*-induced damage. *Bioorganic & Medicinal Chemistry*.

[18] Dragicevic N., Copes N., O'Neal-Moffitt G. (2011). Melatonin treatment restores mitochondrial function in Alzheimer's mice: a mitochondrial protective role of melatonin membrane receptor signaling. *Journal of Pineal Research*.

[19] Re R., Pellegrini N., Proteggente A., Pannala A., Yang M., Rice-Evans C. (1999). Antioxidant activity applying an improved ABTS radical cation decolorization assay. *Free Radical Biology & Medicine*.

[20] Amato K. R., Yeoman C. J., Kent A. (2013). Habitat degradation impacts black howler monkey (*Alouatta pigra*) gastrointestinal microbiomes. *The ISME Journal*.

[21] van de Merwe J. P., Stegeman J. H. (1985). Binding of Coprococcus comes to the Fc portion of IgG. A possible role in the pathogenesis of Crohn's disease?. *European Journal of Immunology*.

[22] Helgeland L., Brandtzaeg P. (2000). Development and function of intestinal B and T cells. *Microbial Ecology in Health and Disease*.

[23] Moore W. E. C., Holdeman L. V. (1974). Human fecal flora: the normal flora of 20 Japanese Hawaiians. *Journal of Applied Microbiology*.

[24] Santoru M. L., Piras C., Murgia A. (2017). Cross sectional evaluation of the gut-microbiome metabolome axis in an Italian cohort of IBD patients. *Scientific Reports*.

[25] Ouwehand A., Isolauri E., Salminen S. (2002). The role of the intestinal microflora for the development of the immune system in early childhood. *European Journal of Nutrition*.

[26] Matijašić M., Meštrović T., Perić M. (2016). Modulating composition and metabolic activity of the gut microbiota in IBD patients. *International Journal of Molecular Sciences*.

[27] Loh G., Blaut M. (2012). Role of commensal gut bacteria in inflammatory bowel diseases. *Gut Microbes*.

[28] Gophna U., Sommerfeld K., Gophna S., Doolittle W. F., Veldhuyzen Van Zanten S. J. O. (2006). Differences between tissue-associated intestinal microfloras of patients with Crohn's disease and ulcerative colitis. *Journal of Clinical Microbiology*.

[29] Van de Merwe J. P., Schmitz P. I. M., Wensinck F. (1981). Antibodies to Eubacterium and Peptostreptococcus species and the estimated probability of Crohn's disease. *Epidemiology & Infection*.

[30] Tyler A. D., Kirsch R., Milgrom R., Stempak J. M., Kabakchiev B., Silverberg M. S. (2016). Microbiome heterogeneity characterizing intestinal tissue and inflammatory bowel disease phenotype. *Inflammatory Bowel Diseases*.

[31] Seril D. N., Liao J., Yang G. Y., Yang C. S. (2003). Oxidative stress and ulcerative colitis-associated carcinogenesis: studies in humans and animal models. *Carcinogenesis*.

[32] Dull B. J., Salata K., Van Langenhove A., Goldman P. (1987). 5-Aminosalicylate: Oxidation by activated leukocytes and protection of cultured cells from oxidative damage. *Biochemical Pharmacology*.

[33] Antolín I., Obst B., Burkhardt S., Hardeland R. (1997). Antioxidative protection in a high-melatonin organism: The dinoflagellate Gonyaulax polyedra is rescued from lethal oxidative stress by strongly elevated, but physiologically possible concentrations of melatonin. *Journal of Pineal Research*.

[34] Pentney P. T., Bubenik G. A. (1995). Melatonin reduces the severity of dextran‐induced colitis in mice. *Journal of Pineal Research*.

